# Evolution of Ethical Principles in the Practice of Pharmaceutical Medicine From a UK Perspective

**DOI:** 10.3389/fphar.2019.01525

**Published:** 2020-01-15

**Authors:** Thomas Morris, Joshua M. Brostoff, Peter David Stonier, Alan Boyd

**Affiliations:** Faculty of Pharmaceutical Medicine, Royal College of Physicians, London, United Kingdom

**Keywords:** pharmaceutical medicine, professionalism, ethical standards, biomedical research, ethical guidance

## Abstract

Pharmaceutical medicine has evolved to be a distinct medical scientific discipline over time. Pharmaceutical medicine has distinctive features related to complex innovative medicines development activities in an often commercially focused competitive environment. This sometimes uneasy mix of professionalism and commercialisation demands of its medical and scientific researchers alike, a focus on strict adherence to ethical standards, guidelines, practices and behaviors in the interest of delivering new, effective, high-quality lifesaving and life-enhancing medicines quickly and reliably to patients in need. To support the speciality, codes of ethical standards and practices have been developed, with several being recently updated. These various codes are outlined in this paper along with relevant historical perspectives and interrelationship with concepts of professionalism. Reflecting the longer history of pharmaceutical medicine as a speciality in the UK and experience of the authors, there is a focus on the UK for the historical perspectives.

## Background

The last five decades has witnessed an increased involvement of the medical profession in the development, introduction and maintenance of medicines. Alongside this has come a greater recognition of the multi-disciplinary nature of the development of medicines, as well as increased regulatory oversight of the processes and procedures involved. In relatively recent times, pharmaceutical medicine has evolved as a medical scientific discipline dedicated to the discovery, development, evaluation, registration, monitoring and the medical aspects of marketing of medicines (Stonier et al., 2007). In 1976 the Royal Colleges of Physicians of Edinburgh, Glasgow and London established the first Diploma in Pharmaceutical Medicine to be gained by examination after a 2-year training course for pharmaceutical physicians. Despite having physicians working for pharmaceutical companies, contract research organizations and regulatory agencies worldwide during this period, there has been limited awareness of the discipline by many academic and national medical associations, contributing to a slow recognition of pharmaceutical medicine as a distinct medical specialty. A pharmaceutical physician is a trained expert on the medical aspects of research, development, evaluation, registration, safety monitoring, and marketing of medicines in the best interests of patients.

### Professional Organisations

There is vigorous debate about what characterizes a professional group or profession, but the following factors are generally regarded as the most important among various authors: a) the possession of abstract specialized knowledge; b) a high degree of individual autonomy; c) authority/influence over customer groups and subordinate occupational groups; d) a degree of altruism; e) a distinction from other occupational groups by higher status and higher pay (Greenwood, 1957; Hashimoto, 2006; Saks, 2012). Professions also are largely self-regulating in the approach they take to ensure that members acquire and maintain the skills and knowledge necessary to perform their role. It is recognized that individual professionals often lose a degree of autonomy when they are employed by large organizations or in government agencies; degrees of authority and influence are also likely to be diminished in such settings. These hindering factors for the professional can also come about from new government regulations and demands of third-party payers that restrict autonomy and influence. Such hindering factors may be more common for pharmaceutical physicians compared to patient-facing clinicians and perhaps argue for the greater need of these individuals to be supported by professional organizations.

The International Federation of Associations of Pharmaceutical Physicians and Pharmaceutical Medicine (IFAPP) was created in 1975 and currently has some 30 affiliated national professional membership associations, representing around 7,000 pharmaceutical physicians and other biomedical professionals involved in medicines development; with the incorporation of non-physician groups being a relatively recent development. IFAPP is a non-profit organisation with the mission to “advance Pharmaceutical Medicine by enhancing the knowledge, expertise and skills of pharmaceutical physicians and other professionals involved in all scientific disciplines involved in the discovery, development, processing and usage of medicines as well as experimental and clinical research worldwide, leading to the availability and appropriate use of medicines for the benefit of patients and society”.

In the UK, the Faculty of Pharmaceutical Medicine (FPM) was founded in 1989 as a faculty of the three Royal Colleges of Physicians of the UK. It is a professional membership organisation and standard-setting body, with around 1,500 members and fellows, a quarter based outside the UK. There are currently some 150 pharmaceutical physicians undergoing post-graduate pharmaceutical medicine specialty training (PMST) through the FPM, and over 360 have achieved the outcome Certificate of Completion of Training (CCT) since pharmaceutical medicine was recognized as a medical specialty in 2002. This certificate allows them to be entered onto the specialist register of the UK General Medical Council.

## Ethical Codes and Guidance

One characteristic of a profession, especially a healthcare-related profession, is that the behavior of its members is guided by a formal code of ethics. Pharmaceutical medicine is unusual in embracing two parallel but converging ethical frameworks: one concerning individual medical practice, and the other regarding the physician’s role in clinical research.

Ethical codes concerning individual clinical practice have evolved since the time of Hippocrates. The principal purpose of such guidance is to assure the best interests of patients and members of the public. These interests must be protected above the need for income and advancement for healthcare practitioners themselves.

The development of clinical research ethics has followed a different path owing to its comparatively recent appearance. The ethics of clinical research have developed reactively, in response either to scandal or to novel scientific techniques (Faden and Beauchamp, 1986; Berg et al., 2001; Emanuel and Grady, 2007; Kimmelman, 2009). Thankfully, the modern trend is towards proactive modification of ethical codes. No longer are they merely to prevent a repeat of sins from the past but are instead forward-looking.

Such codes ideally support individual members of a profession to maintain high professional standards. To achieve this they share two common features:

- They must be prescriptive to a large degree. A descriptive or analytic moral framework provides insufficient support for an individual practitioner. Endorsing and proscribing certain behaviors gives clarity.
- They are based on mid-level ethical principles such as those proposed by Beauchamp and Childress (Holm, 1995; Beauchamp, 2003). Such principles allow for common ground when discussing ethical dilemmas with other stakeholders.

Medical practice ethics for clinicians and researchers come from three levels: global, national, and local. Global codes of ethics include those of the World Medical Association and CIOMS. Most national bodies also produce ethical guidelines for physicians engaging in patient care and in research with ethics codes for individual clinical practice now embedded in national medical regulatory bodies. In the UK the General Medical Council (a regulatory body) and the British Medical Association (a trade union) both provide guidelines on ethics. Many regulatory guidelines and practices (e.g. CIOMS) have worldwide applicability and irrespective of whether or not the concerned physicians have a specialist registration in pharmaceutical medicine.

Local institutional guidelines—often informal or even unstated—are equally important. While many research institutes have policies and procedures relating to ethical research, the tone of research in a hospital, pharmaceutical company, or research facility may be set by a community of peers or by a few senior researchers. Institutional pressure on individual decision-making is well recognized. “Breaking ranks” with established tradition can have unpleasant repercussions.

The shortcoming of the traditional approach is that most codes focus on the moral guidelines of a single profession. However, most pharmaceutical physicians now work in, and are highly reliant on, cross-functional, inter-disciplinary teams to deliver their ultimate goals. The new Ethics Framework from IFAPP seeks to address this shortcoming and applies to scientists as well as physicians working in pharmaceutical clinical development and research arenas. IFAPP recommends that education in ethics should be integrated into the various training courses provided for individuals in these fields. Achievement of professional excellence can then be fostered, and self-identity and professional aspirations supported. 

### Why a Specific Code of Ethics for Pharmaceutical Physicians?

There are many codes of practice for healthcare professionals, differing by country, by culture, and by role. Berwick and others have called for a unified code of ethics for everybody involved in healthcare, but it seems that such a code may be too broad to help the individual practitioner (Berwick et al., 1997). Many ethical duties apply to all doctors, but we consider that there are two main reasons why a specific code of conduct for pharmaceutical physicians is warranted: their regular involvement in clinical trials of experimental medicines, and their work in commercially focused organizations.

Many clinicians engage in research at some point in their careers, but for only a few is it the mainstay of their job. Pharmaceutical physicians, however, are almost certain to have regular involvement with clinical trials. This can range from early phase trials with experimental medicines, through to late phase confirmatory and post-authorization studies. This heavy involvement in clinical trials is a distinguishing feature of pharmaceutical medicine.

Pharmaceutical physicians also have different communities from other clinicians. Clinician-researchers in hospitals or academia are swimming in the same ethical waters as their peers and co-workers. They are also likely to have been mentored by another clinician-researcher and to have implicitly bought in to a shared set of values. In contrast, pharmaceutical physicians often work independently from other clinicians, and are embedded within cross-functional teams.

Pharmaceutical physicians frequently have a business element to their job, or work for pharmaceutical companies that rely on commercial success. They can find their role involving conflicts between commercial imperatives and ethical decisions. Many pharmaceutical physicians also work outside of hospitals or academic centers where a code of medical ethics is part of the institutional culture. In these latter cases a physician’s research work remains connected to their clinical practice, and both these elements of work are embedded within an institutional framework that is highly focused on the patient and on biomedical research. There is no over-arching need to make a profit and hence less need to focus on applied or use-inspired research.

For a pharmaceutical physician in a commercial organization a clash of values can take many forms, perhaps most clearly where for purely commercial reasons a company discontinues development of a drug that seems highly promising. A physician can indeed advocate for continued development, and here ethical and pro-social arguments compete directly with a broader financial interest. Physicians in industry tend to have little freedom to choose or direct the research in an organization. We note of course that the work of a physician in any setting is often constrained by environmental financial factors: for reasons of cost, some procedures or medications may not be available in a particular country, region, or hospital. However in these cases the ethical argument is around resource allocation, and any trade-off is against the well-being of other patients rather than the profit of a private company.

Pharmaceutical physicians working for government agencies can also face organizational pressures. Regulatory agencies have strong cultures, are often part of other governmental agencies, and there can be implicit or explicit pressure to approve or reject new medicines. The physician is acting on behalf of the state rather than on behalf of an individual patient. There are also close ties between the regulator and the industry itself. As the House of Commons Health Committee noted, “the relationship between the industry and the MHRA is naturally close. There are regular interchanges of staff, common policy objectives, agreed processes, shared perspectives and routine contact and consultation” (United Kingdom House of Commons Health Committee, 2005).

### IFAPP International Ethics Framework

The new IFAPP International Ethics Framework for Pharmaceutical Physicians and Medicines Development Scientists was formerly known as the International Code of Ethical Conduct for Pharmaceutical Physicians, published in 2003. It was revised in 2016 considering the rapidly changing and increasingly complex scientific environment of medicines innovation and need to adapt ethical conduct to scientific progress. The present revision aims to provide an ethical framework for both pharmaceutical physicians and medicines development scientists about how to manage pro-actively difficult, and frequently new situations responsibly before they become major problems (Kerpel-Fronius et al., 2018). The new environment has led to re-organization of medicines development teams, with closer, more integrated involvement of specialized basic research groups. Advanced therapies including gene and cell therapies, or tissue engineering cannot be applied in clinical practice without fully integrating basic scientists into the development and treatment teams.

Pharmaceutical physicians have always collaborated with other members of research and development teams as well as with regulatory, marketing and other colleagues in the pharmaceutical industry or regulatory agencies. It is important to address the ethical responsibilities of the entire medicines development team including both basic research and clinical research experts.

We note that pharmaceutical companies are increasingly including ethical practice in their values and mission statements. There is a global shift towards increasing transparency and promotion of ethical practice within the pharmaceutical industry itself (Shaw and Whitney, 2016).

Pharmaceutical physicians and medicines development scientists must always remain aware that the interests of patients and their own employers are best served by an objective scientific attitude and a rigorous ethical approach. IFAPP recognizes that this may place practicing pharmaceutical physicians and scientists in positions that demand considerable determination, and an ethical code can play a vital role in enabling them to reconcile their professional lives with their personal values.

The ethical framework recognizes that some ethical issues are only relevant to pharmaceutical physicians, and an increasing number of challenges must be faced jointly with scientists. For both groups it should be their primary objective to ensure the protection of the dignity, rights, needs and interests of the research participants.

The bioethical principles of Beauchamp and Childress—respect for autonomy, beneficence, non-maleficence and justice—provide a foundation for determining the ethical behavior of both physicians and scientists working in medicines research. They form a basis for balanced ethical judgment in conflict situations, although it is evident that experts in medicines development weigh these principles differently according to the circumstances. Additional ethical principles of relevance to research and development activities include vulnerability, subsidiarity and solidarity, as well as consideration of the duties to the society regarding objective-setting and appropriate research conduct.

The IFAPP Ethics Framework intends to provide an educational background to guide both pharmaceutical physicians and medicines development scientists through their day-to-day deliberations and decision-making whether they practice within a company, contract research organization, academic department, regulatory authority, or work on ethics committees or as independent consultants.

### CIOMS Ethical Guidelines for Biomedical Research

The fourth version of the CIOMS Ethical Guidelines for Biomedical Research was published in 2016 (CIOMS). The scope of the 2002 Guidelines was broadened from “biomedical research” to “health-related research” and the guidelines are now entitled ‘International Ethical Guidelines for Health-related Research Involving Humans’. Despite some debate about the way the guidelines were developed, they are broad and inclusive (Schuklenk, 2017; Schuklenk, 2017).

As also noted by IFAPP several developments had taken place since the last version of their Ethical Guidelines, among them:

- The Declaration of Helsinki had been updated to the 7th revision (2013).
- A heightened emphasis on the importance of translational research.
- A need to clarify what counts as fair research in low and middle-income country settings.
- A greater emphasis on community engagement in research.
- An awareness that exclusion of potentially vulnerable groups in many cases has resulted in weaknesses in the evidence base.
- The increase in the research use of big data.

Following extensive evidence retrieval and synthesis processes, international consultation and peer review the latest CIOMS guidelines form a comprehensive reference tool. The document is over 100 pages and includes 25 guidelines with commentary plus appendices providing guidance on items to be included in a protocol and essential information to be provided to prospective research participants.

### FPM Guiding Principles and Good Pharmaceutical Medical Practice

The FPM Guiding principles were developed in 2010 and updated in 2014 to provide an ethical framework for medical practitioners practicing in the field of pharmaceutical medicine, whether in industry, regulatory bodies or an academic environment (Bragman et al., 2010). These were derived from the original publication and full report published in 2006 (Bickerstaffe et al., 2006; Bickerstaffe et al., 2006). The document clarified that pharmaceutical physicians are bound by the same ethical standards that apply to all doctors. However, their work leads to some very specific ethical considerations that may not be fully explored in ethical codes based on clinical practice. It clearly placed the doctor’s duties to the wider public and the protection of patients and research participants ahead of responsibilities to an individual employer. It also emphasizes the importance of medical leadership in promoting ethical principles and accountability in decision-making.

In 2013, the UK General Medical Council published the Good Medical Practice (GMP) document (General Medical Council, 2013). This forms the core guidance for all registered doctors in the UK and centers on four Domains. 1: Knowledge, skills and performance; 2: Safety and quality; 3: Communication, partnership and teamwork; 4: Maintaining trust. It is supported by a range of explanatory guidance covering fundamental ethical principles that most doctors will use every day e.g. Consent and Confidentiality. There is guidance that may be more relevant to doctors working in certain specialties, or about specific situations that not all doctors will encounter in their career.

The focus of the GMC guidelines is on clinical specialties; pharmaceutical medicine, as highlighted earlier, does bring very specific ethical considerations which may not be fully explored in ethical codes based in clinical medicine. Hence the FPM established a working group to evaluate the needs of pharmaceutical physicians, and later built on the GMC document to create Good Pharmaceutical Medicine Practice (GPMP) in 2008 and updated November 2014, tailored towards the pharmaceutical physician, and explaining how requirements in GMP should be interpreted for those working in pharmaceutical medicine (Good Pharmaceutical Medical Practice, 2014).

GPMP is being reviewed again with an updated document expected in 2020. The Faculty will need to decide if the older Guiding principles document is now redundant as a separate document and should be withdrawn. This is not straightforward as the Guiding Principles were designed foremost to guide those working in pharmaceutical medicine, while GPMP arises from broader medical codes. The underlying principles that guide the protection of patients and research participants, namely; Individuals Come First, Professional integrity and Confidentiality, are completely in line with GMP requirements, however the Guiding Principles goes further in some specific areas. Examples include the need for training in medical ethics and international good clinical practices (GCPs) and promotion of these principles by leadership and example, as well as seeking to raise standards of ethical conduct amongst colleagues and fellow staff. Regulatory Work and Marketing Work are drawn out with examples, e.g. ensuring proposed labeling of a medicinal product accurately reflects the clinical trial data, there is openness and transparency in publication and sharing of research results, and awareness of possible business and commercial pressures. Other specific points are for promotion of all medicines to be supervised by pharmaceutical physicians and be based on objective, ongoing assessment of all the available information. Promotion must be in accord with the labeling and not involve the use of undue pressures or inducements of any nature on healthcare workers to prescribe a product.

Although a document based on GPMP has more impact and authority for UK registered doctors, the Guiding Principles have the great merits of being relatively short, developed specifically for Pharmaceutical Medicine, and relevant globally wherever FPM fellows and members work.

### Embedding Ethical Attitudes in Pharmaceutical Physicians: Clear Communication, Training and Support

A significant challenge is how to embed codes of ethics and standards into the way that professionals think and behave on a day-to-day basis.

Clarity is an important feature of ethical codes. The content must be communicated to those who require it, and length and ease of reading of documents can be substantial barriers to their reading and understanding.

The FPM has made ethical practice a part of its curriculum. All those who train in pharmaceutical medicine must, over the course of their PMST programme, demonstrate integrity and ethical practice. This begins the process of new pharmaceutical physicians developing an ethical grounding.

The FPM sought to develop the original guiding principles as a short document, refer to them in Faculty and other meetings, send printed copies to members and send periodic links to electronic copies available on the website. Alongside the Code and supporting Continuing Professional Development, the FPM has launched a support network and commitment to support those working in the pharmaceutical medicine arena to make the best decisions relating to ethics, probity and integrity.

### Future Considerations

We expect ethical principles related to pharmaceutical medicine and health research in general to continue to evolve with time. With the future advancements in treatment approaches and paradigms this seems inevitable and particular ethical issues will surround areas such as advanced therapies utilizing cell and gene therapies and regenerative medicines as these receive an ever increasing number of approvals. Therapies based on gene editing techniques will also bring their own ethical issues which as a specialty, we will have to face. The use of ‘Big Data’, AI and Real World Data will also require special considerations as far as ethics is concerned; questions such as who actually owns and who should own these data, and how consent is obtained to use such data will need to be debated.

In conclusion, the last fifty years has seen great strides in the development of codes of ethical standards and practices plus support structures for the speciality of pharmaceutical medicine and medicines development. It seems clear that ethical issues and principles will continue to be ever present and continue to evolve. Newer entrants into pharmaceutical medicine should also be encouraged to participate in this evolution. We support the sharing of the principles of medical ethics at undergraduate level for future physicians and healthcare scientists.

## Author Contributions

All authors listed have made a substantial, direct and intellectual contribution to the work, and approved it for publication.

## Conflict of Interest

The authors declare that the research was conducted in the absence of any commercial or financial relationships that could be construed as a potential conflict of interest.

The handling editor is currently organizing a Research Topic with one of the authors PS, and confirms the absence of any other collaboration.
